# Suppression of IFN-Induced Transcription Underlies IFN Defects Generated by Activated Ras/MEK in Human Cancer Cells

**DOI:** 10.1371/journal.pone.0044267

**Published:** 2012-09-07

**Authors:** Sherri L. Christian, Dong Zu, Maria Licursi, Yumiko Komatsu, Theerawat Pongnopparat, Dianne A. Codner, Kensuke Hirasawa

**Affiliations:** Division of Biomedical Science, Faculty of Medicine, Memorial University of Newfoundland, St. John’s, Newfoundland, Canada; Virginia Polytechnic Institute and State University, United States of America

## Abstract

Certain oncolytic viruses exploit activated Ras signaling in order to replicate in cancer cells. Constitutive activation of the Ras/MEK pathway is known to suppress the effectiveness of the interferon (IFN) antiviral response, which may contribute to Ras-dependent viral oncolysis. Here, we identified 10 human cancer cell lines (out of 16) with increased sensitivity to the anti-viral effects of IFN-α after treatment with the MEK inhibitor U0126, suggesting that the Ras/MEK pathway underlies their reduced sensitivity to IFN. To determine how Ras/MEK suppresses the IFN response in these cells, we used DNA microarrays to compare IFN-induced transcription in IFN-sensitive SKOV3 cells, moderately resistant HT1080 cells, and HT1080 cells treated with U0126. We found that 267 genes were induced by IFN in SKOV3 cells, while only 98 genes were induced in HT1080 cells at the same time point. Furthermore, the expression of a distinct subset of IFN inducible genes, that included RIGI, GBP2, IFIT2, BTN3A3, MAP2, MMP7 and STAT2, was restored or increased in HT1080 cells when the cells were co-treated with U0126 and IFN. Bioinformatic analysis of the biological processes represented by these genes revealed increased representation of genes involved in the anti-viral response, regulation of apoptosis, cell differentiation and metabolism. Furthermore, introduction of constitutively active Ras into IFN sensitive SKOV3 cells reduced their IFN sensitivity and ability to activate IFN-induced transcription. This work demonstrates for the first time that activated Ras/MEK in human cancer cells induces downregulation of a specific subset of IFN-inducible genes.

## Introduction

Oncolytic virus specifically replicate in cancer cells, but not in normal cells, by exploiting differences in the intracellular environment of tumor cell that promotes abnormal cell growth [1], [2], [3], [4]. Constitutive activation of Ras signaling was originally reported to be used by oncolytic reovirus to increase its replicative ability [5]. Following the discovery of reovirus oncolysis, other viruses, such as wild type herpes simplex virus (HSV) [6], vesicular stomatitis virus (VSV) [4], influenza virus (delNS1 strain) [7], adenovirus (VAI mutant) [8], poliovirus [9], and Newcastle disease virus [10] were found to similarly exploit activated Ras signaling pathway for oncolysis. Ras is a membrane bound GTP-binding protein that acts as a molecular switch to activate downstream pathways to regulate proliferation, differentiation and transformation [11]. In the canonical Ras pathway, GTP-bound Ras activates its downstream mediator, the Raf kinase. Activated Raf then phosphorylates and activates the MEK1/2 kinases, which phosphorylate and activate the extracellular signal-regulated kinase (ERK) 1 and 2. ERK1/2 can then activate or inhibit transcription factors to promote cell survival and proliferation [12]. Activating mutations of Ras have been found in approximately 30% of all human tumours [13]. Moreover, in the absence of the active mutation of Ras, Ras pathway is often activated by inappropriately activation of its upstream signaling components, such as epidermal growth factor receptor, HER2/NEU and Src [14].

Multiple cellular mechanisms that underlie the Ras dependent viral oncolysis have been identified. Inhibition of the antiviral double-strand RNA-activated protein kinase (PKR) by Ras was originally described as a major mechanism for oncolytic virus replication in tumor cells [5], [6]. It has also been shown that activated Ras promotes the uncoating and release of oncolytic reovirus which increases the production of progeny viruses [15]. Ras activation also enhances the efficiency of cap-independent translation of oncolytic poliovirus [9]. Furthermore, we and another group have reported that activation of the Ras pathway can prevent effective activation of type I interferon (IFN) anti-viral response in human cancer cells and mouse fibroblast cells [16], [17], [18], suggesting that the defect of IFN response induced by activated Ras is one of the common mechanisms of viral oncolysis.

IFNs are secreted cytokines that have multiple effects in the body including anti-viral, anti-proliferative and immunomodulatory roles. As such, IFNs are used in the treatment of viral diseases such as hepatitis C virus infection, treatment of cancer and multiple sclerosis. IFN binds to the IFN-α receptor (IFNAR) [19] leading to the activation of two tyrosine kinases, Janus kinase 1 (Jak1) and tyrosine kinase 2 (Tyk2) that are associated with the IFNAR [20], [21]. Jak1 and Tyk2 then phosphorylate signal transducer and activator of transcription (STAT) 1 and STAT2, which then associate with the DNA binding protein IFN regulatory factor 9 (IRF9), to form a heterotrimeric transcription factor termed IFN-stimulated gene factor 3 (ISGF3) [22], [23]. Binding of ISGF3 to the IFN-stimulated response element (ISRE) in the promoters of IFN-inducible genes induces the expression of hundreds of genes collectively known as IFN-stimulated genes (ISG), many with anti-viral, anti-proliferative and immunomodulatory functions [24]. However, the effectiveness of IFN can be limited by anti-IFN proteins encoded by viral genomes or by endogenous cellular suppressors regulating IFN signaling [21].

Previously, we demonstrated that an IFN sensitive virus, VSV, was capable of replicating in NIH3T3 cells with activated Ras/MEK while IFN prevented infection of control NIH3T3 cells [16]. Noser et. al. [25] also reported that inhibition of Ras/MEK in human cancer cell lines restored antiviral responses induced by IFN. These studies clearly demonstrate that Ras/MEK activation underlies IFN impairment in cancer cells. In a follow-up study, we found that activated Ras/MEK suppressed transcription of STAT2, which is one of the essential IFN signaling components [17]. IFN-mediated protection against virus infection was only partially restored in RasV12 transformed NIH3T3 cells with overexpression of STAT2. However, when the Ras/MEK pathway was inhibited by the MEK inhibitor U0126 in RasV12 cells, IFN was as effective in inducing an antiviral response as in vector control cells. Therefore, it is unlikely that the downregulation of STAT2 expression is the sole mechanism involved in the Ras/MEK mediated IFN suppression. Here, to further identify the mechanism of Ras-mediated inhibition of the IFN response, we analyzed the involvement of the Ras/MEK pathway in regulating IFN-induced transcription in human cancer cell lines.

## Results

### Sensitivity of Human Cancer Cell Lines to the IFN-induced Antiviral Response

**Figure 1:**
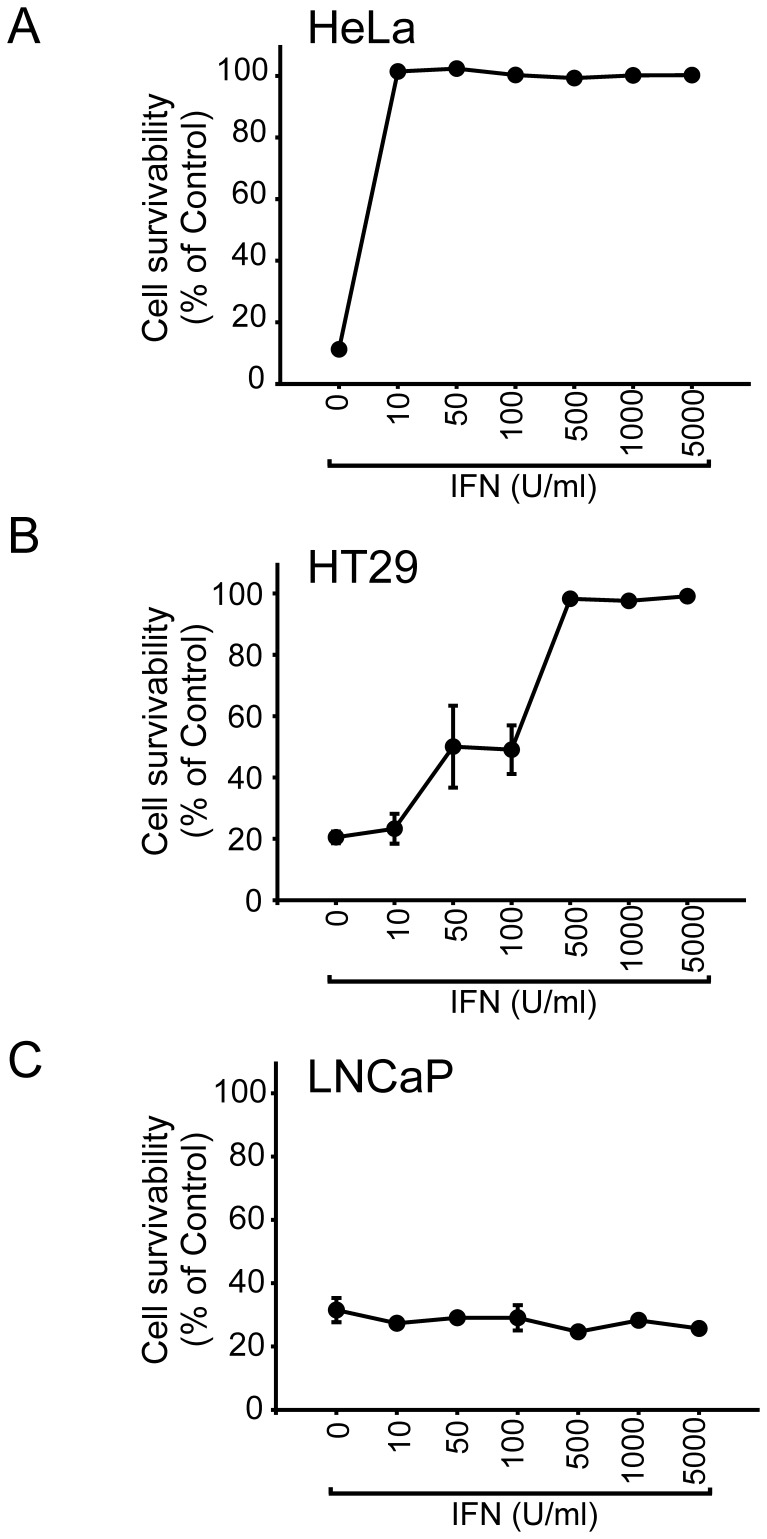
Representative profiles of IFN sensitive, moderately resistant and completely resistant cell lines. IFN sensitive (A), moderately resistant (B) and completely resistant cell lines (C) were identified by pretreating cells with IFN (0, 10, 50, 100, 500, 1000 and 5000 U/ml) for 16 hours and then challenged with VSV at a MOI of 1 for 24 hours. Cell viability was determined using crystal violet staining and expressed as average percentage compared to the uninfected control wells (n = 3 wells). One representative experiment is shown.

First, we selected 16 cancer cell lines derived from different types of tumor (3 breast, 1 cervical, 4 colon, 1 fibrosarcoma, 2 melanoma, 3 ovarian and 2 prostate cell lines) and measured their responsiveness to the anti-viral effect induced by IFN. The cells were treated with IFN (0, 10, 50, 100, 500, 1,000 and 5,000 U/ml) for 16 hours and then challenged with VSV at a multiplicity of infection (MOI) of 1 for 24 hours. The overall level of oncolysis was evaluated using crystal violet staining. Based on the effective dose (cytopathic effect (CPE) 50) of IFN, which is defined as the effective IFN concentration that elicits a 50% protection against VSV infection, the cell lines were divided into the following three groups: IFN sensitive: cell lines with CPE50 less than 10 U/ml, IFN moderately resistant: cell lines with CPE50 between 10 to 5,000 U/ml, and IFN completely resistant: cell lines with CPE50 above the maximum concentration of IFN we tested (5,000 U/ml) (Fig. 1). As shown in Table 1, three cell lines (HeLa, SKBR3 and SKOV3) were sensitive to IFN while 9 cell lines (A375, DLD-1, HT29, HTB129, HT1080, MCF-7, MDAH, MDA-MB468 and SW48) showed moderate resistance to IFN treatment. In contrast, 4 cell lines (DU145, HCT116, LNCaP and PA-1) did not respond to IFN within the concentration range of IFN examined.

**Table 1:** Sensitivity of human cancer cell lines to IFN as measured by antiviral assay.

Sensitive	Moderately Resistant	Completely Resistant
(IFN_CPE50_ a <10 U/ml)	(IFN_CPE50_ 10–5000 U/ml)	(IFN_CPE50_>5000 U/ml)
HeLa	A375 (ED50: 1695 U/ml)	DU145
SKBR3	DLD-1 (ED50: 40 U/ml)	HCT116
SKOV3	HT29 (ED50: 51 U/ml)	LNCaP
	HTB129 (ED50: 28 U/ml)	PA-1
	HT1080 (ED50: 219U/ml)	
	MCF7 (ED50: 46 U/ml)	
	MDAH (Ed50: 114 U/ml)	
	MDA-MB468 (ED50: 27 U/ml)	
	SW48 (Ed50: 3587 U/ml)	

a IFN concentration that elicits a 50% protection against VSV infection based on analysis of cytopathic effects (CPE).

### Restoration of IFN Sensitivity by MEK Inhibition in IFN Moderately or Completely Resistant Cancer Cell Lines

**Figure 2:**
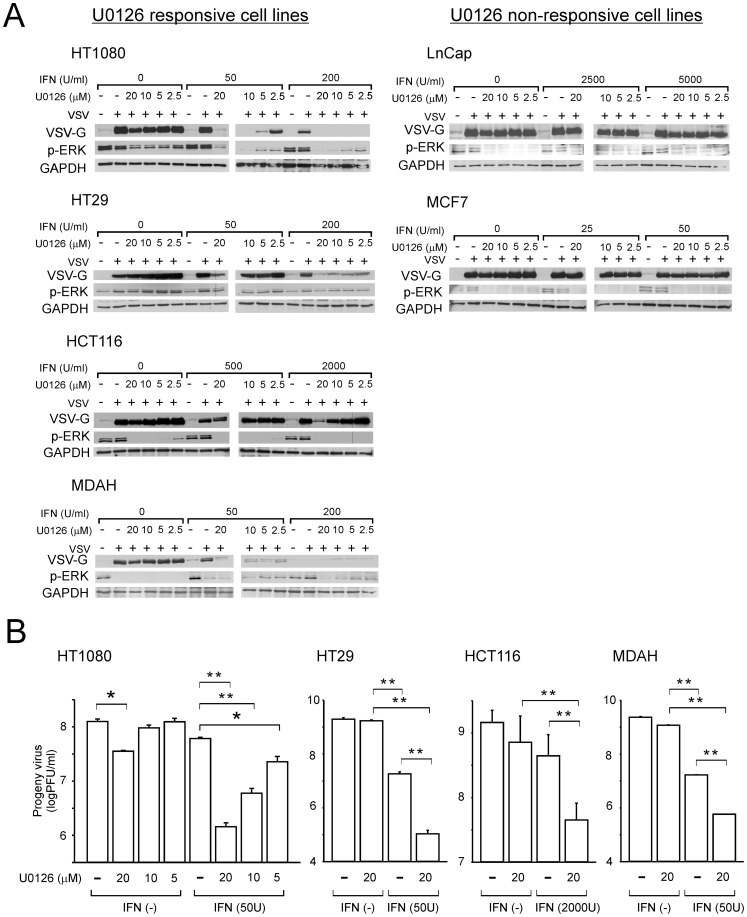
Effect of U0126 treatment on the anti-viral IFN response in moderately resistant and completely resistant cell lines. (A) Cell lines were infected with VSV (MOI = 1) for 24 hours after treatment with IFN (0–5000 U/ml) with or without U0126 (0–20 µM) for 16 hours. Western blot analysis was used to detect viral protein (VSV-G) levels, the level of phosphorylated ERK (p-ERK) with GAPDH used as a loading control. The samples were analyzed on two membranes simultaneously using identical conditions for incubation and detection. One representative experiment out of 3 is shown. (B) Viral progeny production was determined after infection with VSV (MOI = 1) for 24 hours following treatment with IFN (50 or 2000 U/ml) and with U0126 (0, 5, 10 or 20 µM) for 16 hours.

We next determined whether the activation of the Ras/MEK pathway reduces IFN-induced antiviral response in the IFN moderately or completely resistant cancer cell lines. IFN sensitivities of the cell lines were examined in the presence of a MEK inhibitor U0126. The cells were treated with IFN (0, 12.5, 25, 50, 200, 500 and 2000 U/ml) and U0126 (0, 2.5, 5, 10 and 20 µM) for 16 hours and then challenged with VSV at a MOI of 1 for 24 hours. The infection was qualitatively evaluated by western blot analysis of the viral VSV-G protein. The effectiveness of U0126 on MEK inhibition was verified by analysis of ERK phosphorylation, the primary target of MEK [13]. MEK inhibition increased the sensitivity of 10 cancer cell lines to IFN (A375, DLD-1, DU145, HCT116, HT1080, HT29, HTB129, MDA468, MDAH and PA-1) (U0126 responsive cell lines), but had no effect in 3 cancer cell lines (LnCap, MCF7 and SW48) (U0126 non-responsive cell lines) (Fig. 2 and Fig. S1). For example, VSV replicated in HT1080 cells in the presence of IFN (50 and 200 U/ml) while VSV replication was inhibited in cells treated with the same amount of IFN in combination with U0126 treatment (Fig. 2A). HT1080 cells also had slightly reduced VSV infection in the presence of 20 µM U0126 alone. Similarly, IFN-induced antiviral response was restored in HT29, HCT116 and MDAH cells when the Ras/MEK pathway is inhibited by U0126. In contrast, we did not observe the restoration of the IFN-induced antiviral response at any combination of concentrations of U0126 and IFN in LnCap and MCF7 cells suggesting that their IFN resistance is regulated by cellular factors other than activated Ras/MEK pathway. The same analysis was conducted to examine the promotion of IFN-induced antiviral response in the other cell lines (A375, DLD-1, DU145, HTB129, MDA468, PA-1 and SW48) (Fig. S1).

The restoration of antiviral response by MEK inhibition in the four U0126-responsive cell lines was confirmed by progeny virus assay (Fig. 2B). HT1080 cells, infected with VSV, showed a significant reduction in progeny virus production with combined treatment with IFN (50 U/ml) and all concentration of U0126 (5, 10 and 20 µM). Furthermore, U0126 (20 µM) alone showed a modest but statistically significant reduction in viral progeny. In the other cell lines (HT29, HCT116 and MDAH), the combined IFN and U0126 treatment showed a substantial and statistically significant reduction in viral progeny production compared to IFN only or U0126 only treatment indicating increased responsiveness to IFN upon MEK inhibition.

These results indicate that activation of the Ras/MEK pathway suppresses IFN-induced anti-viral activities in some cancer cell lines. We found no correlation between either IFN responsiveness or U0126 responsiveness, and cancer cell types.

### Effect of Ras/MEK Inhibition on IFN-induced Transcription

**Figure 3:**
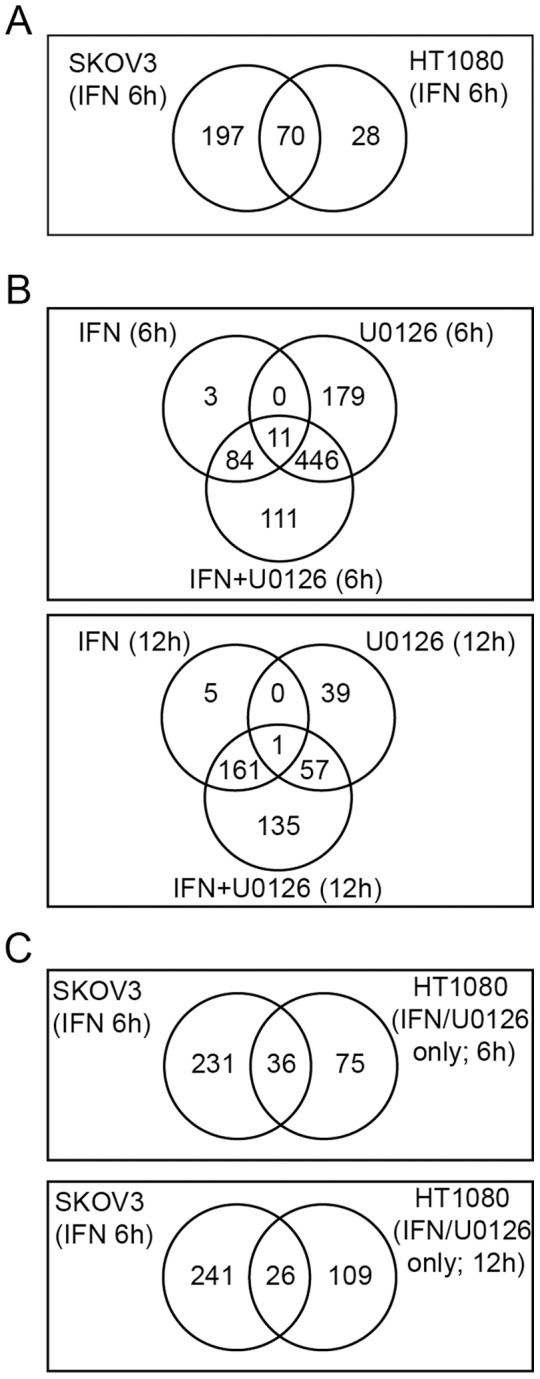
Microarray analysis of IFN inducible genes in IFN sensitive SKOV3 cells, moderately resistant HT1080 cells treated with IFN, U0126 or both. (A) Venn diagrams from DNA microarray analysis showing global suppression of IFN-regulated genes in HT1080 cells compared to SKOV3 cells. Shown are the number of genes significantly upregulated (FDR <0.01) at 6 hours after IFN treatment in the SKOV3 vs. HT1080 cell lines. (B) Venn diagrams showing the number of genes significantly upregulated (FDR <0.01) in HT1080 cells following treatment with IFN, U0126 or both IFN and U0126 for 6 hours or 12 hours as indicated. (C) Venn diagrams comparing genes upregulated by “protective” treatments in each cell type (IFN for SKOV3 cells and IFN/U0126 for HT1080 cells).

To study how activated Ras/MEK suppresses the IFN response in human cancer cells, we conducted global gene expression analysis and identified genes with statistically increased expression compared to the untreated time-matched control (see supporting information [File S1, S2, S3, S4, S5, S6, S7] for complete lists of differentially expressed genes). First, we compared expression of IFN inducible genes in IFN sensitive SKOV3 cells and moderately resistant HT1080 cells (Fig. 3A). Upon IFN stimulation for 6 hours, 267 genes were upregulated in SKOV3 cells while only 98 genes were induced in HT1080 cells. Seventy genes were induced commonly in both SKOV3 and HT1080 cells while 197 IFN inducible genes were upregulated only in SKOV3 cells and 28 IFN inducible genes only in HT1080 cells. These results demonstrate that IFN-induced transcription is suppressed in IFN moderately resistant HT1080 cells compared to IFN sensitive SKOV3 cells.

We then determined whether inhibition of Ras/MEK could change the transcriptional response of HT1080 cells to IFN. IFN only treatment activated transcription of 98 genes at 6 hours and 167 genes at 12 hours while U0126 only treatment induced expression of 636 genes at 6 hours and 97 genes at 12 hours (Fig. 3B). Combined treatment of IFN and U0126 induced transcription of 652 genes at 6 hours and 354 genes at 12 hours. These results demonstrate that activated MEK suppresses IFN-induced transcription in IFN moderately resistant HT1080 cells. Interestingly, we found 111 genes at 6 hours (Table S1) and 135 genes at 12 hours (Table S2) were significantly induced by the combined treatment with IFN and U0126 while either treatment alone did not induce expression, demonstrating true synergistic regulation of gene expression by IFN and Ras/MEK suppression. These genes include mediators of anti-viral function (eg. Apobec3 [26], IFIT2 [27], [28], [29], RSAD2 (viperin) [30], GBP2 [31] and MAP2 [32], [33], [34]), activators of anti-viral signal transduction, (eg. RIGI [35]), regulators of antigen processing and presentation (eg. IFI30 (GILT) [36], BTN3A3 [37] and PSME1 [38]), and regulators of tumourigenesis (eg. MMP7 [39]). Since VSV replicated more than 30 times less efficiently in HT1080 cells treated with IFN and U0126 compared to those treated with IFN only or U0126 only (Fig. 2B), we believe that some of the genes upregulated by the combined treatment in HT1080 cells (111 genes at 6 hours 135 genes at 12 hours) have a significant anti-viral function. We then compared the genes induced in HT1080 by the combined treatment of IFN and U0126 to those induced in SKOV3 cells by IFN only treatment to further narrow down which genes may be the essential genes necessary for a protective anti-viral IFN response (Fig. 3C). We found that 36 genes were commonly induced at 6 hours, and 26 genes at 12 hours, in the two experimental groups (Table S3).

**Figure 4:**
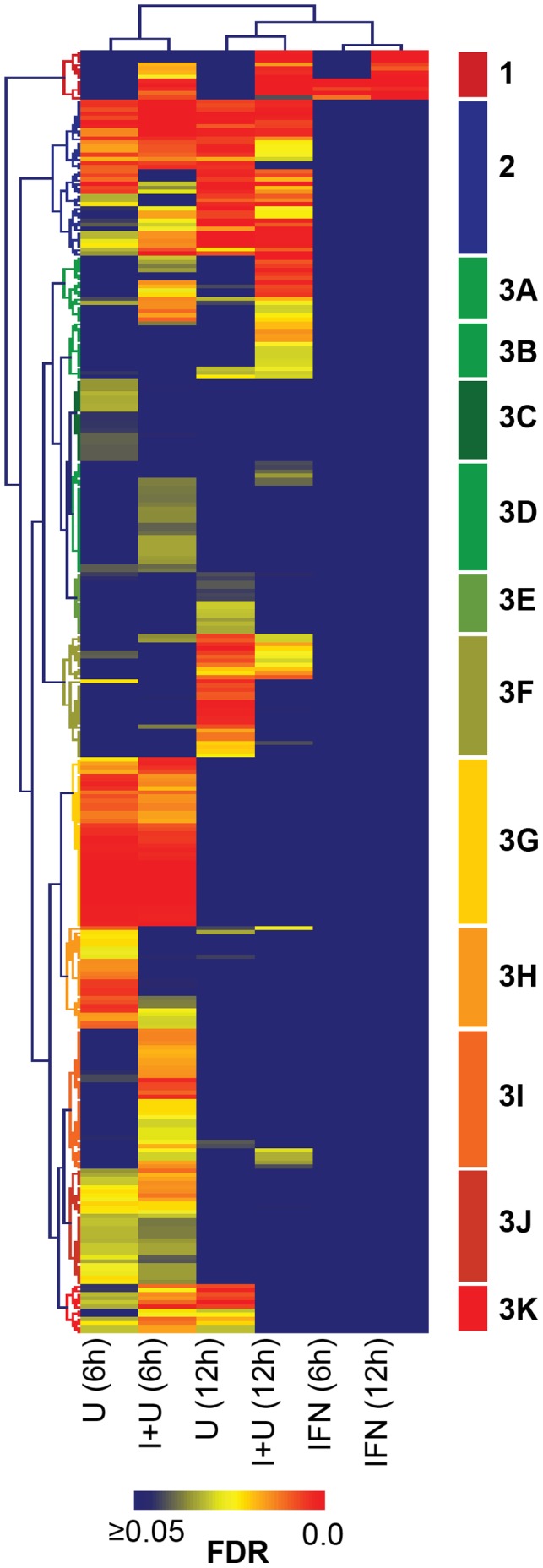
Gene ontology (GO) analysis of genes differentially expressed by IFN (I), U0126 (U) or combined treatment (I+U). Genes with significantly upregulated or downregulated expression levels compared to control were analyzed for over-representation of GO category compared to that observed in the genome. Significantly over-represented GO categories (FDR <0.05) are shown with the highest FDR = 0 shown in red and categories with FDR ≥0.05 or absent shown in blue.

Gene ontology (GO) analysis was performed to determine the biological process associated with the genes identified as significantly changed in HT1080 cells treated with IFN only, U0126 only, and both IFN and U0126 (Table 2 and Fig. 4). Treatment with U0126 only or both IFN and U0126 for 6 hours clustered together indicating that these treatments changed the expression of genes with similar functions (GO over-representation). Similarly, the 12 hour treatments with U0126 alone or the combined treatment resulted in the most similar GO over-representation groups. IFN-only treatment groups (6 and 12 hours) clustered together likely because a relatively small number of GO categories changed when compared to the other treatment groups. Overall, genes fell into 312 GO categories that were associated with 3 GO over-representation clusters where cluster 3 could be further divided into 11 sub-clusters. Clusters 1, 3A, 3B, 3D and 3I showed increased GO over-representation in response to combined U0126 and IFN treatment compared to either treatment alone. These clusters included GO categories associated with NF-κB signaling, anti-viral response, immune response, regulation of apoptosis, chemokine signaling, cellular development, and metabolism. In contrast, clusters 3C, 3E, 3F, 3H and 3K had reduced GO-over-representation categories upon combined treatment, indicating that the addition of IFN stimulation resulted in the loss of gene regulation of genes belonging to GO categories associated with cell motility, DNA repair and cell division. Therefore, MEK inhibition changes the types of genes that can be induced by IFN treatment in addition to increasing the overall number of genes induced.

**Table 2:** Gene ontology analysis of genes that are differentially expressed with combined IFN and U0126 treatment compared to the single treatments.

GO Cluster #	IFN+U0126 response compared to single treatments		GO Cluster Description
	**6h**	**12h**	
1	↑ a	–	Response to virus, NF-κB signaling
2	–	–	Regulation of MAP kinase activity, Transcription factor activity, TGF-β signaling, angiogenesis, anti-apoptosis
3A	↑	↑	Anti-viral response, inflammatory response
3B	↑	↑	Cytokine stimulus, regulation of phosphorylation
3C	↓	–	Cell cycle, metabolism, proliferation
3D	↑	–	Regulation of SMAD TF, Apoptosis, Cell development, response to hypoxia
3E	–	↓	Cell growth, chemotaxis
3F	–	↓	Cell proliferation, chemotaxis,
3G	–	–	DNA replication & repair
3H	↓	–	DNA replication & repair
3I	↑	–	Chemokine signaling & migration, Cell development & differentiation
3J	–	–	Skeletal system development, DNA replication, NK cell activation
3K	–	↓	Cell motility

a ↑: increased representation, ↓: decreased representation, – no change.

Together, these analyses indicate that the Ras/MEK pathway suppresses expression of ISGs involved in antiviral response at multiple levels including detection of pathogen associated patterns, activation of the anti-viral signaling cascade and direct anti-viral effector genes.

### Validation of Restoration of IFN-induced Gene Expression by MEK Inhibition

**Figure 5:**
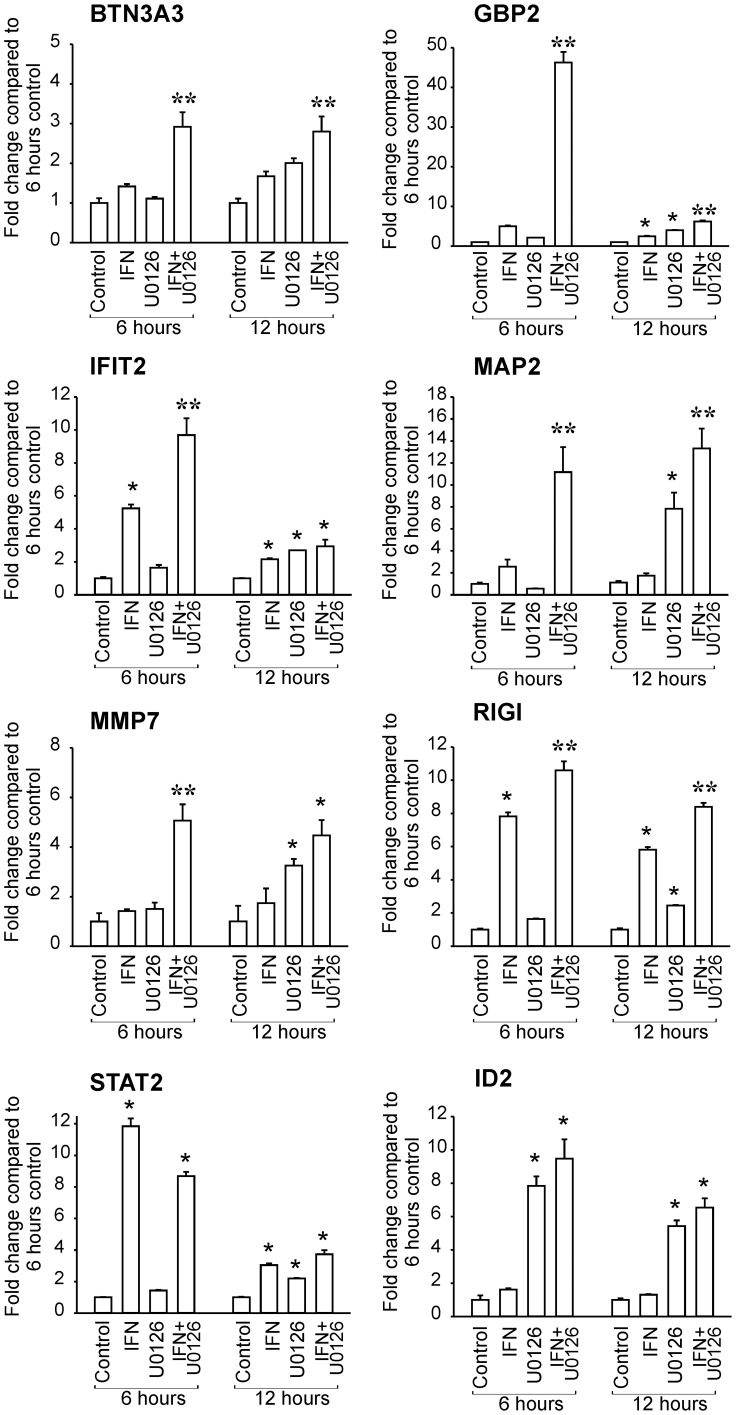
Validation of changes in gene expression by quantitative RT-PCR. The level of gene expression in HT1080 cells left untreated, treated with IFN (50 U/ml), with U0126 (20µM) or with IFN and U0126 for 6 hours or 12 hours was determined by quantitative RT-PCR. The relative expression level was calculated compared to the 6 hour untreated control after normalization against GAPDH expression levels. (n = 3, * P<0.01 compared to time-matched control, ** P<0.05 compared to all other groups).

Quantitative RT-PCR (RT-qPCR) was conducted to confirm the changes in gene expression observed in the microarray analysis using HT1080 cells treated with IFN and/or U0126 (Fig. 5). Eight genes (IFIT2, GBP2, MAP2, RIGI, STAT2, BTN3A3, MMP7 and ID2) were selected based on their biological functions and gene expression changes in response to IFN and/or U0126 treatment. IFIT2, GBP2, MAP2 and MMP7 were upregulated only in HT1080 cells treated with both U0126 and IFN at 6 hours while gene induction was observed in IFN only and U0126 only groups at the later time point (12 hours). BTN3A3 was induced by the combined treatment at both time points, but not by U0126 only or IFN only treatment. Furthermore, Ras/MEK inhibition by U0126 was capable of promoting gene expression of RIGI and STAT2 at 12 hours in the absence of IFN, as described previously [17], [40]. We found that ID2, which is not an IFN-inducible gene, was upregulated only by U0126 treatment and the combined treatment, but not by IFN treatment. Most of the changes in gene expression were confirmed by RT-qPCR, but due to the difference in sensitivity and statistical power of the two different techniques, not all gene changes were identified as statistically significant in both analyses. For example, RIGI was found to be induced in HT1080 cells by IFN only treatment using RT-qPCR, but not in the microarray analysis.

### Effects of Ras activation on IFN-induced Transcription in IFN Sensitive SKOV3 Cells

**Figure 6:**
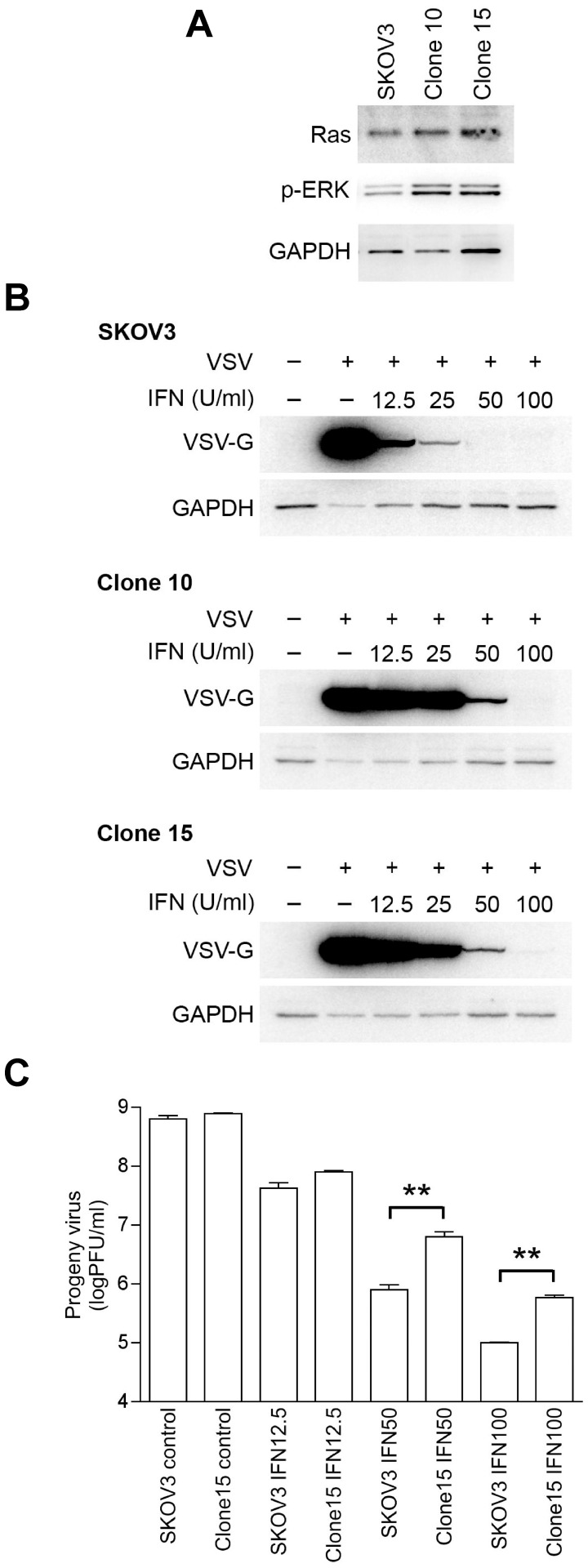
Effect of Ras transformation on the efficiency of viral infection in IFN sensitive SKOV3 cells. (A) Western blot analysis using anti-Ras, phosphorylated ERK or GAPDH antibody to determine Ras/MEK activation in control SKOV3 and Ras transformed SKOV3 (clone 10 and 15). Control SKOV3 and Ras transformed SKOV3 cells were treated with IFN (0, 12.5, 25, 50 and 100 U/ml), and then challenged with challenged with VSV (MOI of 1). Infection was evaluated by (B) western blot analysis for VSV-G protein and GAPDH at 24 hours infection and by (C) progeny virus assay at 48 hours after infection.

**Figure 7:**
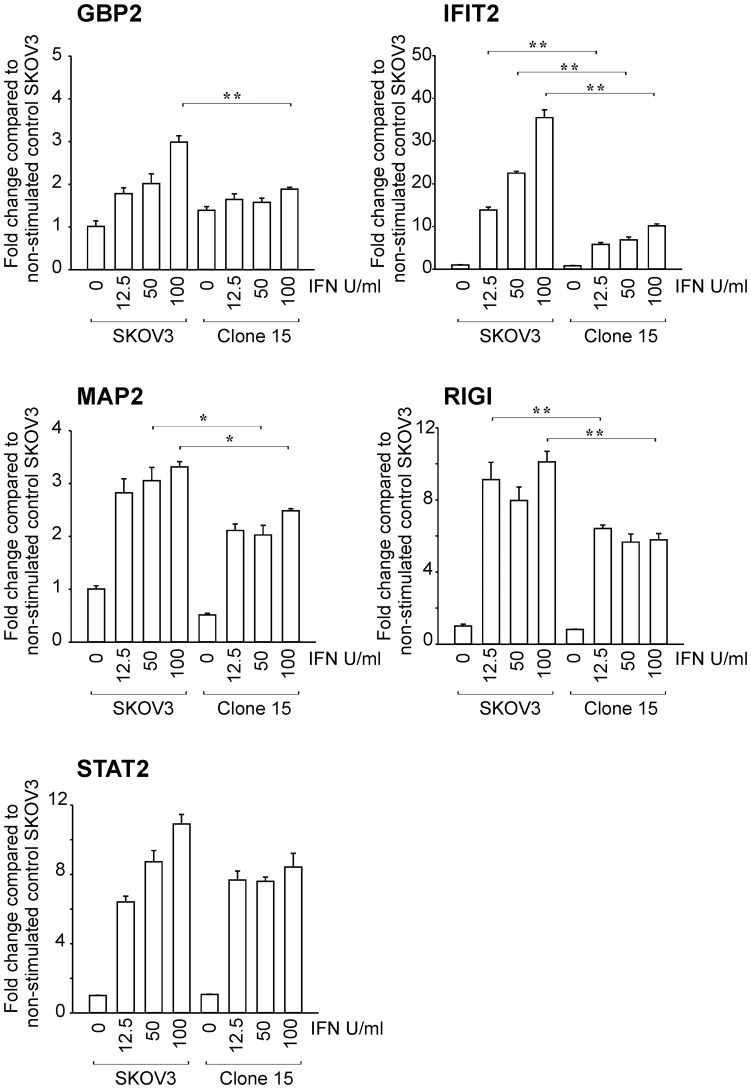
Activation of IFN-induced transcription in control SKOV3 and Ras-transformed SKOV3 cells. The expression of GBP2, IFIT2, MAP2, RIGI and STAT2 in control SKOV3 and Ras-transformed SKOV3 cells (clone 15) at 12 hours after IFN stimulation (0, 12.5, 50 and 100 U/ml) was determined by quantitative RT-PCR. The relative expression level was calculated compared to the untreated control SKOV3 cells after normalization against GAPDH expression levels. (n = 3, * P<0.05 and ** P<0.01 compared to IFN concentration-matched control).

To further examine the suppression of IFN transcription by activated Ras, we generated SKOV3 cells (IFN-sensitive) that express the constitutively active Ras mutant (SKOV3-RasV12; clones 10 and 15). Ras/MEK activation in the mutant cells was confirmed by western blot analysis using anti-phosphorylated ERK, and Ras antibodies (Fig. 6A). Vector control SKOV3 and SKOV3-RasV12 cells were treated with IFN (0, 12.5, 25, 50 and 100 U/ml) for 16 hours and then challenged with VSV at an MOI of 1. Western blot analysis of VSV-G protein at 24 hours after infection demonstrated that VSV clearly replicated more efficiently in Ras-transformed SKOV3 mutants (clone 10 and 15) than in control SKOV3 cells in the presence of IFN (Fig. 6B). We also measured progeny viral levels 48 hours after infection to quantify the degree of viral infection (Fig. 6C). In agreement with the western blot analysis, we found that progeny virus production was significantly higher in the Ras-transformed SKOV3 mutant than in control SKOV3 cells treated with IFN (50 and 100 U/ml). To further determine whether the decrease in IFN sensitivity by the introduction of active Ras is induced by the downregulation of IFN-induced transcription, we examined the changes IFN-induced transcription of 5 genes (GBP2, IFIT2, MAP2, STAT2 and RIGI), which we previously identified to be downregulated by Ras/MEK in HT1080 cells (Fig 5). The induction of GBP2, IFIT2, MAP2 and RIGI was inhibited in the Ras-transformed SKOV3 cells compared to control SKOV3 cells at 12 hours after IFN stimulation while STAT2 induction was not significantly affected (Fig. 7). These results demonstrate that activated Ras signaling suppresses the transcription of certain IFN-inducible genes leading to the decrease in cellular sensitivity to IFN.

## Discussion

As demonstrated previously, the impairment of IFN response in cancer cells is considered one of the common mechanisms for viral oncolysis [3], [10]. Activation of the Ras/MEK pathway has been reported to suppress antiviral responses induced by IFN [4], [16], [17], [25], [40]. In this study, we first surveyed a panel of human cancer cell lines and determined 1) their responsiveness to IFN in producing an anti-viral state and 2) the ability of the MEK inhibitor, U0126, to restore the IFN sensitivity. We found that 13 out of the 16 cell lines tested were moderately or completely resistant to the IFN’s anti-viral response. In 10 out of these 13 cell lines, the sensitivity to IFN was restored upon treatment with the MEK inhibitor. These results suggest that activated Ras/MEK pathway underlies cancer cell resistance to anti-viral effects induced by IFN. To further investigate the underlying mechanism for the suppressive effect of the Ras/MEK pathway on IFN response, we conducted global gene expression analysis to determine IFN-induced transcriptional activities in IFN sensitive SKOV3 and IFN moderately resistant HT1080 cells. We found that there was a substantial reduction in the number of genes induced by IFN in HT1080 cells compared to that in SKOV3 cells (276 genes in SKOV3, 98 genes in HT1080 cells; Fig. 3A). Furthermore, MEK inhibition restored IFN’s ability to activate its transcription in HT1080 cells, as we found that additional 111 genes at 6 hours and 135 genes at 12 hours were expressed in HT1080 cells treated with both IFN and U0126 (Fig. 3B), indicating that activated Ras/MEK suppresses transcription of a group of IFN-induced genes. Finally, we were able to demonstrate that expression of constitutively active Ras in IFN sensitive SKOV3 cells reduced their ability to activate IFN-induced transcription and to establish IFN-induced antiviral response. These results clearly demonstrate that activated Ras/MEK pathway suppresses transcription of certain IFN inducible genes to establish IFN impairment in human cancer cells.

We can hypothesize several possible underlying mechanisms for the widespread suppression of the IFN-induced transcription by the Ras/MEK pathway. 1) Ras/MEK regulates the activity of a transcriptional co-regulator for ISGF3, such as the positive co-regulators IRF1 [41], [42], and Sp3 [43] or the negative co-regulators NF-κB [44], and IFN-consensus sequence binding protein [45]. In this case, IFN-inducible genes that require the up- or down-regulation of a co-regulator for their expression would be suppressed in cells with activated Ras/MEK. 2) Ras/MEK suppresses the basal expression levels of key components of the IFN signaling pathway. Insufficient expression levels of these components leads to the global impairment of IFN induced antiviral response similar to the downregulation of STAT2 expression that we observe in NIH3T3 cells overexpressing constitutively active Ras [17]. 3) The expression of IFN-inducible genes can be suppressed by epigenetic mechanisms. For example, the interaction of STAT2 with BRG1 [46], a key component of the ATP-dependent chromatin remodeling SWI1-SNF2 complex [46], may alter the expression of a subset of IFN-regulated genes. While regulation of BRG1 by Ras has not been demonstrated, downregulation of the related protein, Brm1, has been reported [47]. Similarly, Ras-mediated regulation of DNA methylation [48] or histone modification [49], if targeted to IFN-regulated genes, could globally suppress their expression. 4) Lastly, the Ras/MEK pathway may activate or upregulate negative regulators of the IFN pathway such as SOCS [50] or PIAS [51]. These negative regulators may suppress transcription of the IFN inducible genes identified to be downregulated by Ras/MEK in this study.

Analysis of the biological processes represented in the gene sets revealed that the numbers of biological processes affected by IFN and U0126 was higher than either U0126 or IFN alone. In addition, the number of different GO categories represented was higher at 6 hours compared to 12 hours in either U0126 alone or the combined treatment suggesting that long-term stimulation limits the number of biological processes that can be carried out by the genes that are expressed. As expected, IFN was able to regulate genes known for the response to virus and NF-κB signaling, however, the combined treatment increased the representation of more anti-viral GO categories as well as genes important for cytokine regulation. Furthermore, compared to U0126 alone treatment, combined U0126 and IFN treatment decreased the representation of genes responsible for DNA replication and repair, cell cycle regulation and cell motility. Overall, the combined treatment augmented the anti-viral/inflammatory gene expression response in concert with decreased representation of genes promoting successful cell division.

Interestingly, a subset of genes was induced commonly by the two protective treatments, IFN treatment in SKOV3 cells and combined IFN and U0126 treatment in HT1080 cells (Table S3). These genes included the C14orf159 gene that has been recently shown to have broad anti-viral function, and the MOV10 and IFI44 genes shown to have more restricted anti-viral activity [52]. Similarly, members of the anti-retroviral APOBEC3 [53] and TRIM [53] gene families were induced by both protective treatments. IFIT2 has recently been demonstrated to have anti-viral function [27], [28], [29] and anti-proliferative function [54]. Furthermore, IFIT2 interacts with microtubules [27] suggesting that IFIT2 may associate with IFN-upregulated MAP2 to interfere with virion assembly and/or transport by regulating microtubule dynamics [32], [33], [34]. In addition we also identified critical antiviral genes, such as guanylate binding protein (GBP)-2 [31] and RIGI [35], which are synergistically induced in HT1080 cells treated with U0126 and IFN. Therefore, these data provide further evidence of the importance of these genes for establishing a successful anti-viral response. Future studies will seek to identify the precise roles of these genes, either singly or in combination, for mediating the protective IFN’s anti-viral response and regulating viral oncolysis.

There was no correlation between the tissue origin of human cancer cells used in this study and sensitivity to IFN or the ability of U0126 to restore IFN responsiveness. This suggests that the resistance to IFN by increased activation of the Ras/MEK pathway is acquired independently of the original tissue type. Therefore, tumour type does not appear to be a reliable predictor of the response to combined IFN and MEK inhibition treatment. This is consistent with the fact that oncolytic viruses usually infect a wide range of cancer types. However, since some of the cancer cell lines examined showed such a profound reversal in IFN responsiveness upon MEK inhibition, future studies on signaling components involved in Ras/MEK-mediated impairment of IFN-inducible genes will identify a reliable biomarker of cancer cells to predict their sensitivity against oncolytic virus therapy.

The findings from this study potentially may also have impact in IFN anti-cancer therapy. While IFN therapy has shown significant therapeutic effects on patients with certain types of cancer [55], [56], the therapeutic goals have not been achieved due to the development of cancer cells resistant to IFN treatment [57], [58], [59], [60], [61]. In fact, we confirmed that activation of Ras/MEK also interferes with IFN’s anti-proliferative effects in human cancer cells (data not shown). The growth inhibition by IFN was promoted synergistically in the presence of the MEK inhibitor in U0126-responsive cell lines. Small molecule inhibitors of Ras/MEK have recently progressed to clinical trials of cancer treatment and are proven to have low toxicity *in vivo* [62], [63] Therefore, it is a practical idea to use the combined therapy of IFN with Ras/MEK inhibitors in clinical settings to treat cancer patients.

In summary, we found that activation of the Ras/MEK pathway induces a widespread impairment of IFN-inducible gene expression, resulting in the decreased sensitivity to IFN of human cancer cells. We believe that the downregulation of IFN-inducible transcription by Ras is one of common mechanisms of viral oncolysis among IFN sensitive oncolytic viruses. Furthermore, it may underlie the development of a subpopulation of cancer cells resistant to IFN anti-cancer therapy. Further identification of signaling molecules involved in the Ras/MEK mediated suppression of IFN-induced transcription will improve efficacy and safety of oncolytic viral therapies as well as IFN anti-cancer therapies.

## Materials and Methods

### Cells, Viruses and Reagents

Human cancer cells (A375, DLD-1, DU145, HCT116, HeLa, HT1080, HT29, HTB129, LNCaP, MCF-7, MDAH, MDA-MB468, PA-1, SKBR3, SKOV3 and SW48) and L929 cells, were obtained from the American Type Culture Collection. All cell lines used in this study were maintained in high-glucose DMEM (Invitrogen, Burlington, Ontario, Canada) with 10% FBS (Cansera, Etobicoke, Ontario, Canada). Vesicular stomatitis virus (VSV) (Indiana strain, provided by J.C. Bell, University of Ottawa, Ottawa, Canada) [3] was amplified and titered using L929 cells. Recombinant human IFN-α and U0126 were obtained from AbD Serotec (Raleigh, NC) and Calbiochem (La Jolla, CA), respectively. An antibody to phosphorylated ERK1/2 was obtained from Upstate (Lake Placid, NY), VSV-G protein from Alpha Diagnostic (San Antonio, TX), anti Ras antibody from Cell Signaling Technology (Danvers, MA) and GAPDH from Santa Cruz Biotechnology (Santa Cruz, CA). The activated Ras mutant (H-Ras) construct in the pBABE retroviral vector was generously provided by P.W. Lee (Dalhousie University, Halifax, Canada). The RasV12 vector was transfected into SKOV3 cells using Superfect (Qiagen) and selection of stable transfectants performed in 2 µg/ml puromycin (Invitrogen). Individual clones were selected and expression of constitutively active RasV12 was determined by western blot analysis using anti Ras and phosphorylated ERK1/2 antibody.

### Cell Culture, Virus Infection and Progeny Virus Assay

Cells were plated in 96 well plates (anti-viral assays) or 24 well plates (western blot analysis and progeny viral assay). For the anti-viral assay, the cells were treated with IFN-α (0–5,000 U/ml) for 16 hours and then challenged with VSV at a MOI of 1 PFU/cell. The cells were stained with crystal violet solution (0.5% crystal violet in 10% formalin) for 15 minutes. After washing the plates, the crystal violet staining was extracted with acetic acid and the cell viability was quantified with a spectrophotometer at 590 nm. For western blot analysis and viral progeny assay, the cells were treated with IFN-α (0–500U/ml) and/or U0126 (0–20 µM) for 16 hours and then challenged with VSV at a MOI of 1. The supernatant was harvested for a progeny virus assay. The viral concentrations of supernatants from the triplicate wells were determined by plaque assay as previously described [64]. The cells were washed in PBS and lysed in PBS containing 1% NP-40, 0.5% sodium deoxycholate, 0.1% SDS, 10 mg/ml aprotinin, 100 mg/ml PMSF and 1% phosphatase inhibitor cocktail (Sigma). Protein samples were cleared of debris by centrifugation.

### Western Blot Analysis

The protein concentration was determined by the Bradford method (Bio-Rad, Mississauga, Ontario, Canada). The samples were subjected to 10% SDS-PAGE and transferred to nitrocellulose membranes (Bio-Rad). The membrane was blocked with 5% skim milk in TBS (20 mM Tris and 137 mM NaCl [pH7.3]) containing 0.1% Tween 20 and then incubated with the primary antibodies as listed above. After washing, the membrane was incubated with peroxidase-conjugated goat anti-mouse IgG or anti-rabbit IgG (Santa Cruz), and specific bands were detected by ECL (Amersham, Baie d'Urfe, Quebec, Canada) as described previously [65].

### DNA Microarray Analysis

RNA was isolated from HT1080 cells that had been treated with DMSO control, 20 µM U0126, 50 U/ml IFN-α, or both U0126 and IFN-α for 6 hours or 12 hours and from SKOV3 cells that were treated with 50 U/ml IFN-α for 6 hours using TriZOL (Invitrogen) following the manufacturer’s instructions. To remove genomic DNA contamination, RNA was treated with Turbo DNA-free (Ambion, Austin, TX) according to the manufacturer’s instructions. RNA was shipped to The Centre for Applied Genomics (TCAG, Toronto, Canada) where all further manipulations were carried out. RNA integrity number was determined to be greater than 8.9 for all samples (Agilent 2100 Bioanalyzer, Agilent, Santa Clara, CA). cRNA synthesis, labeling, hybridization and scanning were performed by TCAG. The Affymetrix Human Gene 1.0 ST GeneChip (Affymetrix, Santa Clara, CA) was used to interrogate expression of 28,869 genes with whole-transcript coverage (Gene Expression Omnibus Accession number GSE31019).

Data analysis was performed using Bioconductor 2.6 [66] in R 2.11.0. The data from 3 independent biological replicates were preprocessed using the Oligo package 1.12.2 [67] with the RMA algorithm [68]. Statistically significant changes in gene expression between treatment and control groups were determined using the empirical Bayes moderated t-test from the Linear Models for Microarray Data (LIMMA) package 3.4.3 [69] at a false discovery rate (FDR) of 0.01. Gene ontology (GO) analysis was performed using the High-Throughput GoMiner [70] via the web interface using 1000 permutations, FDR<0.05, UniprotKB, Ensembl, LMP and PDB *Homo sapiens* databases and ALL evidence codes. Clustering based on GO category overrepresentation was performed using Hierarchical clustering with Euclidean distance and average linkage using the Genesis v1.7.1 clustering program [71].

### Quantitative RT-PCR

Quantitative RT-PCR (RT-qPCR) was performed in triplicate using the primers shown in Table S1. Primers were validated using a 5-point, 5-fold dilution series starting with 200 ng cDNA generated from DNase-treated RNA isolated from HT1080 cells treated with both U0126 and IFN for 6 hours using Platinum SYBR Green RT-qPCR kit (Invitrogen, Ontario, Canada) and analyzed on the StepOnePlus qPCR system (Applied Biosystems, Foster City, CA). The cycling conditions were: 50°C for 3 min, 95°C for 5 min followed by 40 cycles of 95°C for 15 sec, 60°C for 30 sec then 40°C for 1 min followed by melt-curve analysis. The absence of non-specific amplification was confirmed by observing a single peak in the melt-curve analysis, confirmation of the expected amplicon size by agarose gel analysis and the absence of primer dimers by the absence of amplification in the no template control wells. The efficiency of each primer set is shown in Table S4 and was taken into consideration when calculating expression levels using a modified relative quantity (ΔΔCt) calculation [72]. Statistical analysis (1-way ANOVA with TUKEY post-doc test) was performed using SigmaPlot.

## Supporting Information

Figure S1 **Effect of U0126 treatment on the anti-viral IFN response in moderately resistant and completely resistant cell lines.** Cell lines were infected with VSV (MOI = 1) for 24 hours after treatment with IFN (0–5000 U/ml) with or without U0126 (0–20 µM) for 16 hours. Western blot analysis was used to detect viral protein (VSV-G) levels, the level of phosphorylated ERK (p-ERK) with GAPDH used as a loading control. The samples were analyzed on two membranes simultaneously using identical conditions for incubation and detection. One representative experiment out of 3 is shown. IFN sensitivity of A375, DLD-1, DU145, HTB 129, MDA468 and PA-1 cells was restored by MEK inhibition (U0126 responsive) while IFN-induced antiviral response was not promoted by U0126 in SW48 cells (U0126 non-responsive).
(PDF)

Table S1 **List of 111 genes significantly upregulated (FDR<0.01) in HT1080 cells treated with both IFN and U016, but not with IFN alone or U0126 alone, for 6 hours.**
(DOCX)

Table S2 **List of 135 genes significantly upregulated (FDR<0.01) in HT1080 cells treated with both IFN and U016, but not with IFN alone or U0126 alone, for 12 hours.**
(DOCX)

Table S3 **Genes significantly upregulated by IFN treatment in SKOV3 cells at 6 h and by combined IFN/U0126 treatment in HT0180 cells but not IFN treatment alone.**
(DOCX)

Table S4 **Sequence, amplicon size and effieciencies of qPCR primers.**
(DOCX)

File S1 **Differentially expressed genes in HT1080 cells treated with IFN for 6 h compared to vehicle treated controls (FDR <0.01).**
(XLS)

File S2 **Differentially expressed genes in HT1080 cells treated with IFN for 12 h compared to vehicle treated controls (FDR <0.01).**
(XLS)

File S3 **Differentially expressed genes in HT1080 cells treated with U0126 for 6 h compared to vehicle treated controls (FDR <0.01).**
(XLS)

File S4 **Differentially expressed genes in HT1080 cells treated with U0126 for 12 h compared to vehicle treated controls (FDR <0.01).**
(XLS)

File S5 **Differentially expressed genes in HT1080 cells treated with IFN and U0126 for 6 h compared to vehicle treated controls (FDR <0.01).**
(XLS)

File S6 **Differentially expressed genes in HT1080 cells treated with IFN and U0126 for 12 h compared to vehicle treated controls (FDR <0.01).**
(XLS)

File S7 **Differentially expressed genes in SKOV3 cells treated with IFN 6 h compared to vehicle treated controls (FDR <0.01).**
(XLS)
