# Supplementary figures and images for: Suppression of IFN-Induced Transcription Underlies IFN Defects Generated by Activated Ras/MEK in Human Cancer Cells
Source: PLoS One. 2012 Sep 7;7(9):e44267. doi: 10.1371/journal.pone.0044267 (PMC3436881; doi:10.1371/journal.pone.0044267)

## U0126 responsive cells

### A375

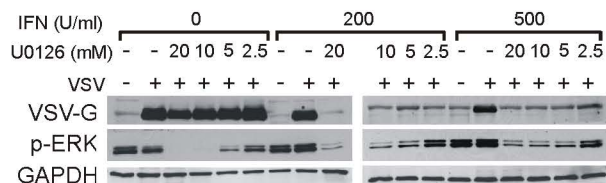

### DLD-1

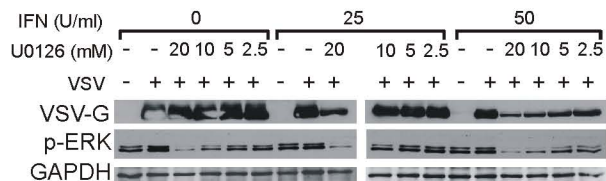

### DU145

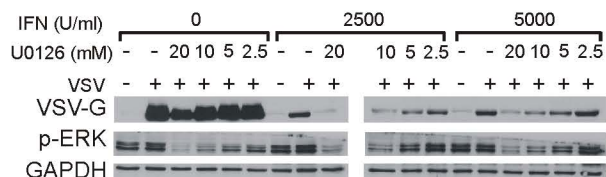

### HTB129

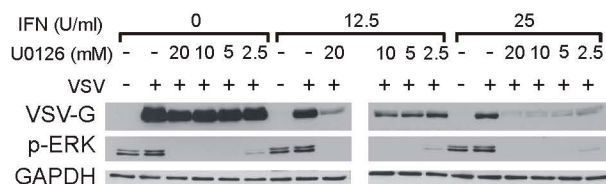

### MDA468

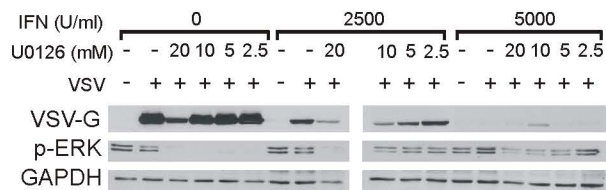

### PA-1

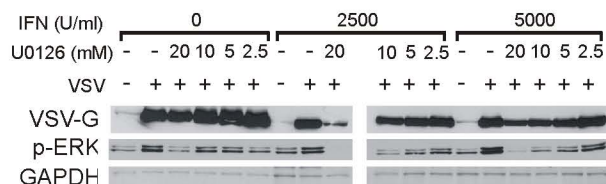

## U0126 non-responsive cells

### SW48

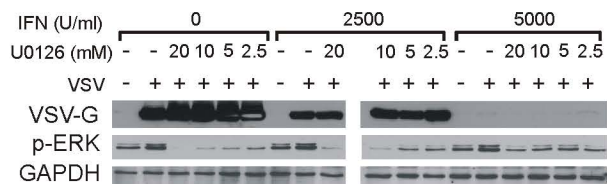

Supplement: Figure S1 — Effect of U0126 treatment on the anti-viral IFN response in moderately resistant and completely resistant cell lines. Cell lines were infected with VSV (MOI = 1) for 24 hours after treatment with IFN (0–5000 U/ml) with or without U0126 (0–20 µM) for 16 hours. Western blot analysis was used to detect viral protein (VSV-G) levels, the level of phosphorylated ERK (p-ERK) with GAPDH used as a loading control. The samples were analyzed on two membranes simultaneously using identical conditions for incubation and detection. One representative experiment out of 3 is shown. IFN sensitivity of A375, DLD-1, DU145, HTB 129, MDA468 and PA-1 cells was restored by MEK inhibition (U0126 responsive) while IFN-induced antiviral response was not promoted by U0126 in SW48 cells (U0126 non-responsive). (PDF) [file pone.0044267.s001.pdf]
