# Supplementary material for: Suppression of IFN-Induced Transcription Underlies IFN Defects Generated by Activated Ras/MEK in Human Cancer Cells
Source: PLoS One. 2012 Sep 7;7(9):e44267. doi: 10.1371/journal.pone.0044267 (PMC3436881; doi:10.1371/journal.pone.0044267)
Supplement: Table S1 — List of 111 genes significantly upregulated (FDR<0.01) in HT1080 cells treated with both IFN and U016, but not with IFN alone or U0126 alone, for 6 hours. (DOCX) [file pone.0044267.s002.docx]

**Table S1: List of 111 genes significantly upregulated (FDR<0.01) in HT1080 cells treated with both IFN and U016, but not with IFN alone or U0126 alone, for 6 hours.**

| **Affymetrix Probe ID** | **Gene Symbol** | **Gene Name** | **Transcript ID** | **Entrez Gene ID** |  |
| --- | --- | --- | --- | --- | --- |
| 8142554 | AASS | aminoadipate-semialdehyde synthase | NM_005763 | 10157 |  |
| 7947947 | AGBL2 | ATP/GTP binding protein-like 2 | NM_024783 | 79841 |  |
| 7921434 | AIM2 | absent in melanoma 2 | NM_004833 | 9447 |  |
| 8073081 | APOBEC3F | apolipoprotein B mRNA editing enzyme, catalytic polypeptide-like 3F | NM_145298 | 200316 |  |
| 8072735 | APOL1 | apolipoprotein L, 1 | NM_003661 | 8542 |  |
| 8121734 | ASF1A | ASF1 anti-silencing function 1 homolog A (S. cerevisiae) | NM_014034 | 25842 |  |
| 7988644 | ATP8B4 | ATPase, class I, type 8B, member 4 | NM_024837 | 79895 |  |
| 7983360 | B2M | beta-2-microglobulin | NM_004048 | 567 |  |
| 8117458 | BTN3A1 | butyrophilin, subfamily 3, member A1 | NM_007048 | 11119 |  |
| 7991512 | C15orf51 | dynamin 1 pseudogene | NR_003260 | 196968 |  |
| 8032608 | C19orf28 | chromosome 19 open reading frame 28 | NM_021731 | 126321 |  |
| 8069505 | C21orf15 | chromosome 21 open reading frame 15 | NR_026755.1 | 54094 |  |
| 8135532 | C7orf53 | chromosome 7 open reading frame 53 | NM_182597 | 286006 |  |
| 8163348 | C9orf84 | chromosome 9 open reading frame 84 | NM_173521 | 158401 |  |
| 8161839 | C9orf95 | chromosome 9 open reading frame 95 | NM_017881 | 54981 |  |
| 8095341 | cdna | Genscan chromosome:GRCh37 | GENSCAN00000026427 | --- |  |
| 8168733 | cdna | cdna:pseudogene chromosome:GRCh37 | ENST00000427866 | --- |  |
| 8007188 | CNP | 2',3'-cyclic nucleotide 3' phosphodiesterase | NM_033133 | 1267 |  |
| 8103389 | CTSO | cathepsin O | NM_001334 | 1519 |  |
| 8101126 | CXCL10 | chemokine (C-X-C motif) ligand 10 | NM_001565 | 3627 |  |
| 8028950 | CYP2G1P | cytochrome P450, family 2, subfamily G, polypeptide 1 pseudogene | NR_040249.1 | 22952 |  |
| 7918008 | DBT | dihydrolipoamide branched chain transacylase E2 | NM_001918 | 1629 |  |
| 8163599 | DFNB31 | deafness, autosomal recessive 31 | NM_015404 | 25861 |  |
| 7902512 | DNAJB4 | DnaJ (Hsp40) homolog, subfamily B, member 4 | NM_007034 | 11080 |  |
| 8076161 | DNAL4 | dynein, axonemal, light chain 4 | NM_005740 | 10126 |  |
| 8059222 | DNPEP | aspartyl aminopeptidase | NM_012100 | 23549 |  |
| 8057045 | FKBP7 | FK506 binding protein 7 | NM_181342 | 51661 |  |
| 8065758 | FLJ38773 | hypothetical protein FLJ38773 | AK096092 | 284808 |  |
| 8041937 | FLJ46838 | hypothetical FLJ46838 protein | ENST00000378305 | 440865 |  |
| 7987145 | FMN1 | formin 1 | NM_001103184 | 342184 |  |
| 8168205 | FOXO4 | forkhead box O4 | NM_005938 | 4303 |  |
| 8007637 | FZD2 | frizzled homolog 2 (Drosophila) | NM_001466 | 2535 |  |
| 8137250 | GIMAP2 | GTPase, IMAP family member 2 | NM_015660 | 26157 |  |
| 7957253 | GLIPR1L2 | GLI pathogenesis-related 1 like 2 | NM_152436 | 144321 |  |
| 8136849 | GSTK1 | glutathione S-transferase kappa 1 | NM_015917 | 373156 |  |
| 8163505 | HDHD3 | haloacid dehalogenase-like hydrolase domain containing 3 | NM_031219 | 81932 |  |
| 8167973 | HEPH | hephaestin | NM_001130860 | 9843 |  |
| 8117343 | HFE | hemochromatosis | NM_000410 | 3077 |  |
| 8179080 | HLA-L /// HLA-A | major histocompatibility complex, class I, L (pseudogene)/ major histocompatibility complex, class I, A | NR_027822 | 3139 /// 3105 |  |
| 8117861 | HLA-L/ HLA-A | major histocompatibility complex, class I, L (pseudogene)/ major histocompatibility complex, class I, A | NR_027822 | 3139 /// 3105 |  |
| 8127743 | HMGN3 | high mobility group nucleosomal binding domain 3 | NM_138730 | 9324 |  |
| 7906400 | IFI16 | interferon, gamma-inducible protein 16 | NM_005531 | 3428 |  |
| 8026971 | IFI30 | interferon, gamma-inducible protein 30 | NM_006332 | 10437 |  |
| 7902553 | IFI44 | interferon-induced protein 44 | NM_006417 | 10561 |  |
| 7929052 | IFIT3 | interferon-induced protein with tetratricopeptide repeats 3 | NM_001031683 | 3437 |  |
| 7969003 | ITM2B | integral membrane protein 2B | NM_021999 | 9445 |  |
| 7910950 | KMO | kynurenine 3-monooxygenase (kynurenine 3-hydroxylase) | NM_003679 | 8564 |  |
| 8025382 | LASS4 | LAG1 homolog, ceramide synthase 4 | NM_024552 | 79603 |  |
| 8005809 | LGALS9 | lectin, galactoside-binding, soluble, 9 | NM_009587 | 3965 |  |
| 7963024 | LMBR1L | limb region 1 homolog (mouse)-like | NM_018113 | 55716 |  |
| 7986428 | LOC400464 | hypothetical protein LOC100288426 | AK127420 | 400464 |  |
| 7945663 | LOC402778 | CD225 family protein FLJ7651 | NM_001170820 | 402778 |  |
| 7954460 | LYRM5 | LYR motif containing 5 | NM_001001660 | 144363 |  |
| 8047926 | MAP2 | microtubule-associated protein 2 | NM_031847 | 4133 |  |
| 7939665 | MDK | midkine (neurite growth-promoting factor 2) | NM_002391 | 4192 |  |
| 7905329 | MLLT11 | myeloid/lymphoid or mixed-lineage leukemia (trithorax homolog, Drosophila); translocated to, 11 | NM_006818 | 10962 |  |
| 7904050 | MOV10 | Mov10, Moloney leukemia virus 10, homolog (mouse) | NM_020963 | 4343 |  |
| 7932067 | ncrna | snRNA | ENST00000363415 | --- |  |
| 8020451 | ncrna | snoRNA | ENST00000363107 | --- |  |
| 8048976 | ncrna | misc_RNA | ENST00000410320 | --- |  |
| 8050801 | ncrna | snoRNA | ENST00000365609 | --- |  |
| 8061073 | ncrna | snRNA | ENST00000364310 | --- |  |
| 8094739 | ncrna | snRNA | ENST00000363585 | --- |  |
| 8097305 | ncrna: | misc_RNA | ENST00000364427 | --- |  |
| 7995926 | NLRC5 | NLR family, CARD domain containing 5 | NM_032206 | 84166 |  |
| 8153609 | PARP10 | poly (ADP-ribose) polymerase family, member 10 | NM_032789 | 84875 |  |
| 7971922 | PCDH9 | protocadherin 9 | NM_020403 | 5101 |  |
| 7970595 | PCOTH/ C1QTNF9B | prostate collagen triple helix /// C1q and tumor necrosis factor related protein 9B | NM_001014442/ NM_001007537 | 542767 / 387911 |  |
| 8144802 | PDGFRL | platelet-derived growth factor receptor-like | NM_006207 | 5157 |  |
| 7935180 | PDLIM1 | PDZ and LIM domain 1 | NM_020992 | 9124 |  |
| 8086185 | PLCD1 | phospholipase C, delta 1 | NM_001130964 | 5333 |  |
| 7996516 | PLEKHG4 | pleckstrin homology domain containing, family G (with RhoGef domain) member 4 | NM_015432 | 25894 |  |
| 7957570 | PLXNC1 | plexin C1 | NM_005761 | 10154 |  |
| 8101701 | PPM1K | protein phosphatase 1K (PP2C domain containing) | NM_152542 | 152926 |  |
| 8149250 | PRAGMIN | homolog of rat pragma of Rnd2 | NM_001080826 | 157285 |  |
| 8067680 | PRIC285 | peroxisomal proliferator-activated receptor A interacting complex 285 | NM_033405 | 85441 |  |
| 7919305 | PRKAB2 | protein kinase, AMP-activated, beta 2 non-catalytic subunit | NM_005399 | 5565 |  |
| 7994647 | PRRT2 | proline-rich transmembrane protein 2 | NM_145239 | 112476 |  |
| 7973564 | PSME1 | proteasome (prosome, macropain) activator subunit 1 (PA28 alpha) | NM_176783 | 5720 |  |
| 7978123 | PSME2 | proteasome (prosome, macropain) activator subunit 2 (PA28 beta) | NM_002818 | 5721 |  |
| 8177938 | PSORS1C1 | psoriasis susceptibility 1 candidate 1 | NM_014068 | 170679 |  |
| 7975284 | RDH12 | retinol dehydrogenase 12 (all-trans/9-cis/11-cis) | NM_152443 | 145226 |  |
| 7920839 | RIT1 | Ras-like without CAAX 1 | NM_006912 | 6016 |  |
| 8053576 | RNF103 | ring finger protein 103 | NM_005667 | 7844 |  |
| 8040080 | RSAD2 | radical S-adenosyl methionine domain containing 2 | NM_080657 | 91543 |  |
| 8084732 | RTP4 | receptor (chemosensory) transporter protein 4 | NM_022147 | 64108 |  |
| 8120981 | scRNA_pseudogene | ncrna_pseudogene | --- | --- |  |
| 8086953 | SHISA5 | shisa homolog 5 (Xenopus laevis) | NM_016479 | 51246 |  |
| 8104930 | SLC1A3 | solute carrier family 1 (glial high affinity glutamate transporter), member 3 | NM_004172 | 6507 |  |
| 8071107 | SLC25A18 | solute carrier family 25 (mitochondrial carrier), member 18 | NM_031481 | 83733 |  |
| 8130565 | snRNA | ncrna | --- | --- |  |
| 8060334 | SOX12 | SRY (sex determining region Y)-box 12 | NM_006943 | 6666 |  |
| 7964360 | STAT6 | signal transducer and activator of transcription 6, interleukin-4 induced | NM_003153 | 6778 |  |
| 8156861 | STX17 | syntaxin 17 | NM_017919 | 55014 |  |
| 7978208 | TINF2 | TERF1 (TRF1)-interacting nuclear factor 2 | NM_012461 | 26277 |  |
| 8136388 | TMEM140 | transmembrane protein 140 | NM_018295 | 55281 |  |
| 8072659 | TOM1 | target of myb1 (chicken) | NM_005488 | 10043 |  |
| 8162729 | TRIM14 | tripartite motif-containing 14 | NM_033220 | 9830 |  |
| 8087485 | UBA7 | ubiquitin-like modifier activating enzyme 7 | NM_003335 | 7318 |  |
| 7949904 | UNC93B1 | unc-93 homolog B1 (C. elegans) | NM_030930 | 81622 |  |
| 8025000 | VMAC | vimentin-type intermediate filament associated coiled-coil protein | NM_001017921 | 400673 |  |
| 8038515 | VRK3 | vaccinia related kinase 3 | NM_016440 | 51231 |  |
| 7976766 | WDR25 | WD repeat domain 25 | NM_001161476 | 79446 |  |
| 8028652 | ZFP36 | zinc finger protein 36, C3H type, homolog (mouse) | NM_003407 | 7538 |  |
| 8139832 | ZNF117 | zinc finger protein 117 | NM_015852 | 51351 |  |
| 8079204 | ZNF197 | zinc finger protein 197 | NM_006991 | 10168 |  |
| 8029399 | ZNF226 | zinc finger protein 226 | NM_015919 | 7769 |  |
| 8028248 | ZNF570 | zinc finger protein 570 | NM_144694 | 148268 |  |
| 7967863 | ZNF605 | zinc finger protein 605 | NM_183238 | 90462 |  |
| 7896703 | --- | Affx control | --- | --- | |
| 8180374 | --- | Unmapped full-length transcript | --- | --- |  |
