# Supplementary material for: Suppression of IFN-Induced Transcription Underlies IFN Defects Generated by Activated Ras/MEK in Human Cancer Cells
Source: PLoS One. 2012 Sep 7;7(9):e44267. doi: 10.1371/journal.pone.0044267 (PMC3436881; doi:10.1371/journal.pone.0044267)
Supplement: Table S2 — List of 135 genes significantly upregulated (FDR<0.01) in HT1080 cells treated with both IFN and U016, but not with IFN alone or U0126 alone, for 12 hours. (DOCX) [file pone.0044267.s003.docx]

**Table S2: List of 135 genes significantly upregulated (FDR<0.01) in HT1080 cells treated with both IFN and U016, but not with IFN alone or U0126 alone, for 12 hours.**

| **Affymetrix Probe ID** | **Gene Symbol** | **Gene Name** | **Transcript ID** | **Entrez Gene ID** |
| --- | --- | --- | --- | --- |
| 8121277 | AIM1 | absent in melanoma 1 | NM_001624 | 202 |
| 8096919 | ALPK1 | alpha-kinase 1 | NM_025144 | 80216 |
| 8073081 | APOBEC3F | apolipoprotein B mRNA editing enzyme, catalytic polypeptide-like 3F | NM_145298 | 200316 |
| 7954055 | APOLD1 | apolipoprotein L domain containing 1 | NM_030817 | 81575 |
| 8113073 | ARRDC3 | arrestin domain containing 3 | NM_020801 | 57561 |
| 8094911 | ATP10D | ATPase, class V, type 10D | NM_020453 | 57205 |
| 8101002 | BTC | betacellulin | NM_001729 /// BC011618 /// ENST00000311727 /// ENST00000395743 | 685 |
| 8117458 | BTN3A1 | butyrophilin, subfamily 3, member A1 | NM_007048 | 11119 |
| 8117435 | BTN3A2 | butyrophilin, subfamily 3, member A2 | NM_007047 | 11118 |
| 7976216 | C14orf159 | chromosome 14 open reading frame 159 | NM_024952 | 80017 |
| 7979721 | C14orf83 | chromosome 14 open reading frame 83 | NM_182526 | 161145 |
| 7990027 | C15orf28 | chromosome 15 open reading frame 28 | NR_026808 | 80035 |
| 8032608 | C19orf28 | chromosome 19 open reading frame 28 | NM_021731 | 126321 |
| 7908178 | C1orf26 | chromosome 1 open reading frame 26 | NM_017673 | 54823 |
| 7953603 | C1S | complement component 1, s subcomponent | NM_201442 | 716 |
| 8069511 | C21orf81 | ankyrin repeat domain 20 family, member A3 pseudogene | NR_027270 | 391267 |
| 8156549 | C9orf3 | chromosome 9 open reading frame 3 | NM_032823 | 84909 |
| 8008113 | CALCOCO2 | calcium binding and coiled-coil domain 2 | NM_005831 | 10241 |
| 8047403 | CASP10 | caspase 10, apoptosis-related cysteine peptidase | NM_001230 | 843 |
| 7930577 | CASP7 | caspase 7, apoptosis-related cysteine peptidase | NM_033340 | 840 |
| 8013479 | CCDC144C/ CCDC144NL/ CCDC144B/ CCDC144A | coiled-coil domain containing 144C/ coiled-coil domain containing 144 family, N-terminal like/ coiled-coil domain containing 144B/ coiled-coil domain containing 144A/ similar to Coiled-coil domain containing 144B | NR_023380/ NM_001004306/ NR_036647.1/ NM_014695.1 | 348254/ 339184/ 284047/ 9720 |
| 7952914 | CCDC77 | coiled-coil domain containing 77 | NM_001130148 | 84318 |
| 8120719 | CD109 | CD109 molecule | NM_133493 | 135228 |
| 7921332 | CD5L | CD5 molecule-like | NM_005894 | 922 |
| 8007188 | CNP | 2',3'-cyclic nucleotide 3' phosphodiesterase | NM_033133 | 1267 |
| 8077123 | CPT1B/ CHKB-CPT1B/ CHKB | carnitine palmitoyltransferase 1B (muscle)/ choline kinase-like, carnitine palmitoyltransferase 1B (muscle) transcription unit/ choline kinase beta | NM_152245/ NM_005198 | 1375/ 386593/ 1120 |
| 7928308 | DDIT4 | DNA-damage-inducible transcript 4 | NM_019058 | 54541 |
| 8046124 | DHRS9 | dehydrogenase/reductase (SDR family) member 9 | NM_005771 | 10170 |
| 7902512 | DNAJB4 | DnaJ (Hsp40) homolog, subfamily B, member 4 | NM_007034 | 11080 |
| 7902043 | DNAJC6 | DnaJ (Hsp40) homolog, subfamily C, member 6 | NM_014787 | 9829 |
| 8059222 | DNPEP | aspartyl aminopeptidase | NM_012100 | 23549 |
| 7924450 | DUSP10 | dual specificity phosphatase 10 | NM_007207 | 11221 |
| 7961371 | DUSP16 | dual specificity phosphatase 16 | NM_030640 | 80824 |
| 7978558 | EAPP | E2F-associated phosphoprotein | NM_0184534 | 55837 |
| 8148333 | EFR3A | EFR3 homolog A (S. cerevisiae) | NM_015137 | 23167 |
| 7971197 | ELF1 | E74-like factor 1 (ets domain transcription factor) | NM_001145353 | 1997 |
| 7899350 | FAM76A | family with sequence similarity 76, member A | NM_152660 | 199870 |
| 8023843 | FBXO15 | F-box protein 15 | NM_152676 | 201456 |
| 8152703 | FBXO32 | F-box protein 32 | NM_058229 | 114907 |
| 7972932 | FLJ41484 | hypothetical LOC650669 | AK092862 | 650669 |
| 7919314 | FMO5 | flavin containing monooxygenase 5 | NM_001461 | 2330 |
| 8170326 | FMR1 | fragile X mental retardation 1 | NM_002024 | 2332 |
| 8154916 | GALT | galactose-1-phosphate uridylyltransferase | NM_000155 | 2592 |
| 7965941 | GLT8D2 | glycosyltransferase 8 domain containing 2 | NM_031302 | 83468 |
| 8117034 | GMPR | guanosine monophosphate reductase | NM_006877 | 2766 |
| 8105463 | GPBP1 | GC-rich promoter binding protein 1 | NM_022913 | 65056 |
| 8126629 | GTPBP2 | GTP binding protein 2 | NM_019096 | 54676 |
| 8167369 | HDAC6 | histone deacetylase 6 | NM_006044 | 10013 |
| 8117343 | HFE | hemochromatosis | NM_000410 | 3077 |
| 7905067 | HIST2H4B/ HIST2H4A | histone cluster 2, H4b/ histone cluster 2, H4a | NM_001034077/ NM_003548 | 554313/ 8370 |
| 7919627 | HIST2H4B/ HIST2H4A | histone cluster 2, H4b/ histone cluster 2, H4a | NM_001034077/ NM_003548 | 554313/ 8370 |
| 8178833 | HLA-DOB | major histocompatibility complex, class II, DO beta | NM_002120 | 3112 |
| 8024660 | HMG20B | high-mobility group 20B | NM_006339 | 10362 |
| 8118310 | HSPA1A/ HSPA1B | heat shock 70kDa protein 1A/ heat shock 70kDa protein 1B | NM_005345/ AK295398 | 3303/ 3304 |
| 8118314 | HSPA1A/ HSPA1B | heat shock 70kDa protein 1A/ heat shock 70kDa protein 1B | NM_005345/ NM_005346 | 3303/ 3304 |
| 8178086 | HSPA1A/ HSPA1B | heat shock 70kDa protein 1A/ heat shock 70kDa protein 1B | NM_005345/ NM_005346 | 3303/ 3304 |
| 8179322 | HSPA1A/ HSPA1B | heat shock 70kDa protein 1A/ heat shock 70kDa protein 1B | NM_005345/ AK295398 | 3303/ 3304 |
| 8179324 | HSPA1A/ HSPA1B | heat shock 70kDa protein 1A/ heat shock 70kDa protein 1B | NM_005345/ NM_005346 | 3303/ 3304 |
| 8146092 | IDO1 | indoleamine 2,3-dioxygenase 1 | NM_002164 | 3620 |
| 8026971 | IFI30 | interferon, gamma-inducible protein 30 | NM_006332 | 10437 |
| 7902553 | IFI44 | interferon-induced protein 44 | NM_006417 | 10561 |
| 7929047 | IFIT2 | interferon-induced protein with tetratricopeptide repeats 2 | NM_001547 | 3433 |
| 8097553 | IL15 | interleukin 15 | NM_000585 | 3600 |
| 7913768 | IL22RA1 | interleukin 22 receptor, alpha 1 | NM_021258 | 58985 |
| 8093230 | KIAA0226 | KIAA0226 | NM_014687 | 9711 |
| 7926679 | KIAA1217 | KIAA1217 | NM_0195906 | 56243 |
| 8124088 | KIF13A | kinesin family member 13A | NM_022113 | 63971 |
| 7980958 | LGMN | legumain | NM_005606 | 5641 |
| 7934920 | LIPA | lipase A, lysosomal acid, cholesterol esterase | NM_001127605 | 3988 |
| 8107066 | LNPEP | leucyl/cystinyl aminopeptidase | NM_175920 | 4012 |
| 8114470 | LRRTM2/ CTNNA1 | leucine rich repeat transmembrane neuronal 2/ catenin (cadherin-associated protein), alpha 1, 102kDa | NM_015564/ AK297332 | 26045/ 1495 |
| 8123893 | MAK | male germ cell-associated kinase | NM_005906 | 4117 |
| 8047926 | MAP2 | microtubule-associated protein 2 | NM_031847 | 4133 |
| 7926821 | MASTL | microtubule associated serine/threonine kinase-like | NM_032844 | 84930 |
| 7951309 | MMP13 | matrix metallopeptidase 13 (collagenase 3) | NM_002427 | 4322 |
| 7951217 | MMP7 | matrix metallopeptidase 7 (matrilysin, uterine) | NM_002423 | 4316 |
| 7915861 | MOBKL2C/ MKNK1 | MOB1, Mps One Binder kinase activator-like 2C (yeast)/ MAP kinase interacting serine/threonine kinase 1 | NM_201403/ NM_145279 | 148932/ 8569 |
| 7904050 | MOV10 | Mov10, Moloney leukemia virus 10, homolog (mouse) | NM_020963 | 4343 |
| 8072039 | MYO18B | myosin XVIIIB | NM_032608 | 84700 |
| 7970388 | ncrna | snRNA | ENST00000411355/ ENST00000411092 | --- |
| 8069517 | ncrna | snRNA | ENST00000411355 | --- |
| 8098261 | ncrna | snoRNA | ENST00000384442 | --- |
| 8162276 | NFIL3 | nuclear factor, interleukin 3 regulated | NM_005384 | 4783 |
| 8137414 | NUB1 | negative regulator of ubiquitin-like proteins 1 | NM_016118 | 51667 |
| 7937940 | OR52K3P | olfactory receptor, family 52, subfamily K, member 3 pseudogene | AF143328 | 390035 |
| 7919699 | OTUD7B | OTU domain containing 7B | NM_020205 | 56957 |
| 7899455 | PHACTR4 | phosphatase and actin regulator 4 | NM_001048183 | 65979 |
| 8108080 | PHF15 | PHD finger protein 15 | NM_015288 | 23338 |
| 7915787 | PIK3R3 | phosphoinositide-3-kinase, regulatory subunit 3 (gamma) | NM_003629 | 8503 |
| 8037794 | PRKD2 | protein kinase D2 | NM_016457 | 25865 |
| 7973564 | PSME1 | proteasome (prosome, macropain) activator subunit 1 (PA28 alpha) | NM_176783 | 5720 |
| 8055697 | RBM43 | RNA binding motif protein 43 | NM_198557 | 375287 |
| 7904830 | RNF115 | ring finger protein 115 | NM_014455 | 27246 |
| 8150186 | RNF122 | ring finger protein 122 | NM_024787 | 79845 |
| 7909661 | RPS6KC1 | ribosomal protein S6 kinase, 52kDa, polypeptide 1 | NM_012424 | 26750 |
| 8101158 | SCARB2 | scavenger receptor class B, member 2 | NM_005506 /// D12676 /// ENST00000264896 /// ENST00000452464 | 950 |
| 8091698 | SHOX2 | short stature homeobox 2 | NM_003030 | 6474 |
| 8081710 | SIDT1 | SID1 transmembrane family, member 1 | NM_017699 | 54847 |
| 7918426 | SLC16A4 | solute carrier family 16, member 4 (monocarboxylic acid transporter 5) | NM_004696 | 9122 |
| 7988426 | SLC30A4 | solute carrier family 30 (zinc transporter), member 4 | NM_013309 | 7782 |
| 7965964 | SLC41A2 | solute carrier family 41, member 2 | NM_032148 | 84102 |
| 7915543 | SLC6A9 | solute carrier family 6 (neurotransmitter transporter, glycine), member 9 | NM_006934 | 6536 |
| 8048926 | SP140L | SP140 nuclear body protein-like | NM_138402 | 93349 |
| 8113491 | STARD4 | StAR-related lipid transfer (START) domain containing 4 | NM_139164 | 134429 |
| 8156861 | STX17 | syntaxin 17 | NM_017919 | 55014 |
| 8097080 | SYNPO2 | synaptopodin 2 | NM_133477 | 171024 |
| 8178977 | TAPBP | TAP binding protein (tapasin) | NM_172208 | 6892 |
| 8180166 | TAPBP | TAP binding protein (tapasin) | NM_172208 | 6892 |
| 8014633 | TBC1D3/ TBC1D3F/ TBC1D3B/ TBC1D3C/ TBC1D3G/ TBC1D3H | TBC1 domain family, member 3/ TBC1 domain family, member 3F/ TBC1 domain family, member 3B/ TBC1 domain family, member 3C/ TBC1 domain family, member 3G/ TBC1 domain family, member 3H | NM_001040282/ NM_032258/ NM_001001418/ NM_001123391/ NM_001123392/ NM_001001417 | 729873/ 84218/ 414059/ 414060/ 654341/ 729877 |
| 8014376 | TBC1D3F/ TBC1D3/ TBC1D3B/ TBC1D3C/ TBC1D3G/ TBC1D3H | TBC1 domain family, member 3F/ TBC1 domain family, member 3/ TBC1 domain family, member 3B/ TBC1 domain family, member 3C/ TBC1 domain family, member 3G/ TBC1 domain family, member 3H | NM_001040282/ NM_032258/ NM_001001418/ NM_001123391/ NM_001123392/ NM_001001417 | 84218/ 729873/ 414059/ 414060/ 654341/ 729877 |
| 8014397 | TBC1D3F/ TBC1D3/ TBC1D3B/ TBC1D3C/ TBC1D3G/ TBC1D3H | TBC1 domain family, member 3F/ TBC1 domain family, member 3/ TBC1 domain family, member 3B/ TBC1 domain family, member 3C/ TBC1 domain family, member 3G/ TBC1 domain family, member 3H | NM_001040282/ NM_032258/ NM_001001418/ NM_001123391/ NM_001123392/ NM_001001417 | 84218/ 729873/ 414059/ 414060/ 654341/ 729877 |
| 8014420 | TBC1D3F/ TBC1D3/ TBC1D3B/ TBC1D3C/ TBC1D3G/ TBC1D3H | TBC1 domain family, member 3F/ TBC1 domain family, member 3/ TBC1 domain family, member 3B/ TBC1 domain family, member 3C/ TBC1 domain family, member 3G/ TBC1 domain family, member 3H | NM_001040282/ NM_032258/ NM_001001418/ NM_001123391/ NM_001123392/ NM_001001417 | 84218/ 729873/ 414059/ 414060/ 654341/ 729877 |
| 8014437 | TBC1D3F/ TBC1D3/ TBC1D3B/ TBC1D3C/ TBC1D3G/ TBC1D3H | TBC1 domain family, member 3F/ TBC1 domain family, member 3/ TBC1 domain family, member 3B/ TBC1 domain family, member 3C/ TBC1 domain family, member 3G/ TBC1 domain family, member 3H | NM_001040282/ NM_032258/ NM_001001418/ NM_001123391/ NM_001123392/ NM_001001417 | 84218/ 729873/ 414059/ 414060/ 654341/ 729877 |
| 8019716 | TBC1D3F/ TBC1D3/ TBC1D3B/ TBC1D3C/ TBC1D3G/ TBC1D3H | BC1 domain family, member 3F/ TBC1 domain family, member 3/ TBC1 domain family, member 3B/ TBC1 domain family, member 3C/ TBC1 domain family, member 3G/ TBC1 domain family, member 3H | NM_001040282/ NM_032258/ NM_001001418/ NM_001123391/ NM_001123392/ NM_001001417 | 84218/ 729873/ 414059/ 414060/ 654341/ 729877 |
| 8017173 | TBC1D3F/ TBC1D3/ TBC1D3B/ TBC1D3P2/ TBC1D3C/ TBC1D3G/ TBC1D3H/ DHX40P1/ LOC100134348 | TBC1 domain family, member 3F/ TBC1 domain family, member 3/ TBC1 domain family, member 3B/ TBC1 domain family, member 3 pseudogene 2/ TBC1 domain family, member 3C/ TBC1 domain family, member 3G/ TBC1 domain family, member 3H/ DEAH (Asp-Glu-Ala-His) box polypeptide 40 pseudogene 1/ similar to TBC1 domain family member 3 (Rab GTPase-activating protein PRC17) (Prostate cancer gene 17 protein) (TRE17 alpha protein) | NM_001040282/ NM_032258/ NM_001001418/ NM_001123391/ NM_001123392/ NR_027486/ NR_002924 | 84218/ 729873/ 414059/ 440452/ 414060/ 654341/ 729877/ 653645/ 100134348 |
| 8019655 | TBC1D3G/ TBC1D3C/ TBC1D3D/ TBC1D3B/ TBC1D3 | TBC1 domain family, member 3G/ TBC1 domain family, member 3C/ TBC1 domain family, member 3D/ TBC1 domain family, member 3B/ TBC1 domain family, member 3 | NM_001040282/ NM_032258/ NM_001001418/ NM_001123391/ NM_001123392/ NM_001001417 | 654341/ 414060/ 727735/ 414059/ 729873 |
| 8009014 | TLK2 | tousled-like kinase 2 | NM_006852 | 11011 |
| 8019622 | TMEM106A / FLJ77644 | transmembrane protein 106A / hypothetical LOC728772 | NM_145041/ BC146974 | 113277 / 728772 |
| 8007483 | TMEM106A/ FLJ77644 | transmembrane protein 106A/ hypothetical LOC728772 | NM_14504/ AK290387 | 113277/ 728772 |
| 8088054 | TMEM110 | transmembrane protein 110 | NM_198563 | 375346 |
| 7958828 | TRAFD1 | TRAF-type zinc finger domain containing 1 | NM_006700 | 10906 |
| 8135064 | TRIM56 | tripartite motif-containing 56 | NM_030961 | 81844 |
| 7983779 | UNC13C | unc-13 homolog C (C. elegans) | NM_001080534 | 440279 |
| 7960544 | VAMP1 | vesicle-associated membrane protein 1 (synaptobrevin 1) | NM_199245 | 6843 |
| 7976766 | WDR25 | WD repeat domain 25 | NM_001161476 | 79446 |
| 7985482 | WHAMM | WAS protein homolog associated with actin, golgi membranes and microtubules | NM_001080435 | 123720 |
| 8021542 | ZCCHC2 | zinc finger, CCHC domain containing 2 | NM_017742 | 54877 |
| 8021546 | ZCCHC2/ C18orf49 | zinc finger, CCHC domain containing 2/ chromosome 18 open reading frame 49 | NM_017742/ BC047606 | 54877/ 400653 |
| 7979757 | ZFYVE26 | zinc finger, FYVE domain containing 26 | NM_015346 | 23503 |
| 8117685 | ZKSCAN3 | zinc finger with KRAB and SCAN domains 3 | NM_024493 | 80317 |
| 7917530 | --- | cDNA DKFZp451C2311 | AL832451 | --- |
| 8021563 | --- | Homo sapiens clone DNA57695 | AY358662 | --- |
| 8068494 | --- | putative peroxisome microbody protein 175.1 | AF050199 | --- |
| 8101699 | --- | Homo sapiens cDNA FLJ3513 | AK092450 | --- |
| 8180374 | --- | Unmapped full-length transcript | --- | --- |
