# Supplementary material for: Suppression of IFN-Induced Transcription Underlies IFN Defects Generated by Activated Ras/MEK in Human Cancer Cells
Source: PLoS One. 2012 Sep 7;7(9):e44267. doi: 10.1371/journal.pone.0044267 (PMC3436881; doi:10.1371/journal.pone.0044267)
Supplement: Table S3 — Genes significantly upregulated by IFN treatment in SKOV3 cells at 6 h and by combined IFN/U0126 treatment in HT0180 cells but not IFN treatment alone. (DOCX) [file pone.0044267.s004.docx]

**Table S3: Genes significantly upregulated by IFN treatment in SKOV3 cells at 6h and by combined IFN/U0126 treatment in HT0180 cells but not IFN treatment alone .**

| **6h** |  | **12h** |  |
| --- | --- | --- | --- |
| **Gene Symbol** | **Entrez ID** | **Gene Symbol** | **Entrez ID** |
| AIM2 | 9447 | APOBEC3F | 200316 |
| APOBEC3F | 200316 | BTN3A1 | 11119 |
| APOL1 | 8542 | BTN3A2 | 11118 |
| BTN3A1 | 11119 | C14orf159 | 80017 |
| BTN3A2 | 11118 | CASP7 | 840 |
| BTN3A3 | 10384 | CNP | 1267 |
| C14orf159 | 80017 | DNPEP | 23549 |
| CNP | 1267 | ELF1 | 1997 |
| CXCL10 | 3627 | GMPR | 2766 |
| DNPEP | 23549 | GTPBP2 | 54676 |
| GIMAP2 | 26157 | IDO1 | 3620 |
| IFI16 | 3428 | IFI30 | 10437 |
| IFI30 | 10437 | IFI44 | 10561 |
| IFI44 | 10561 | IFIT2 | 3433 |
| IFIT3 | 3437 | KIAA1217 | 56243 |
| LGALS9 | 3965 | MAP2 | 4133 |
| MAP2 | 4133 | MOV10 | 4343 |
| MOV10 | 4343 | NUB1 | 51667 |
| NLRC5 | 84166 | PRKD2 | 25865 |
| PARP10 | 84875 | RPS6KC1 | 26750 |
| PPM1K | 152926 | SP140L | 93349 |
| PRIC285 | 85441 | TRAFD1 | 10906 |
| PSME2 | 5721 | ZCCHC2 | 54877 |
| RSAD2 (cig5) | 91543 | ZFYVE26 | 23503 |
| RTP4 | 64108 | ncrna:snoRNA | --- |
| SHISA5 | 51246 | Unmapped transcript (ID 8180374) | --- |
| SLFN5 | 162394 |  |  |
| TMEM140 | 55281 |  |  |
| TRAFD1 | 10906 |  |  |
| TRIM14 | 9830 |  |  |
| TRIM6 | 117854 |  |  |
| UBA7 | 7318 |  |  |
| UNC93B1 | 81622 |  |  |
| ncrna:misc_RNA | --- |  |  |
| ncrna:snRNA | --- |  |  |
| Unmapped transcript (ID 8180374) | --- |  |  |
