# Supplementary material for: Suppression of IFN-Induced Transcription Underlies IFN Defects Generated by Activated Ras/MEK in Human Cancer Cells
Source: PLoS One. 2012 Sep 7;7(9):e44267. doi: 10.1371/journal.pone.0044267 (PMC3436881; doi:10.1371/journal.pone.0044267)
Supplement: Table S4 — Sequence, amplicon size and effieciencies of qPCR primers. (DOCX) [file pone.0044267.s005.docx]

**Table S4: Sequence, amplicon size and effieciencies of qPCR primers**

| **Gene name** | **5’ primer** | **3’ primer** | **Amplicon size^a^** | **Efficiency (%)^b^** |
| --- | --- | --- | --- | --- |
| BTN3A3 | TCCCTCTTCTTGGTCCAGCTGCT | CACCATGGCCAGGATGGGCC | 84 | 109.97 |
| Ddx58 (RigI) | AGACCACATCCCAAGCCAAAG | ACATGGATTCCCCAGTCATGG | 98 | 85.33 |
| GAPDH | ATCTTCTTTTGCGTCGCCAG | ACGACCAAATCCGTTGACTCC | 82 | 106.02 |
| GBP2 | TTTCACCCTGGAACTGGAAG | TGCACAACCGAGGATCATTA | 125 | 90.84 |
| ID2 | ATCCCCCAGAACAAGAAGGT | ATAGTGGGATGCGAGTCCAG | 98 | 123.47 |
| IFIT2 | ATTGCACTGCAACCATGAGTG | TCCCTCCATCAAGTTCCAGGT | 95 | 99.82 |
| MAP2 | TCCTGCACCCTTTGGTGTTGCA | GGACCCACCCCCACAAACTCC | 237 | 99.53 |
| MMP7 | TGGAATGTTAAACTCCCGCGT | GCCCCACATGTTTAAAGCCTT | 199 | 98.13 |
| STAT2 | GGAATCAGGCATGTGTCCCTT | TTCACCTCTCACCCCAATGGA | 81 | 113.74 |

^a^ Predicted amplicon size using Primer-Blast (NCBI).

^b^ Efficiency determined using a 5-point, 5-fold dilution series starting with 200 ng cDNA.
